# Eucalyptus oil poisoning: two case reports

**DOI:** 10.1186/s13256-019-2260-z

**Published:** 2019-11-04

**Authors:** Abraham M. Ittyachen, Georgie Rajan George, Meera Radhakrishnan, Yetin Joy

**Affiliations:** Department of Medicine, M.O.S.C Medical College & Hospital, Kolenchery, Ernakulam District, Kerala State - 682311 India

**Keywords:** Eucalyptus oil, Poisoning, Seizures

## Abstract

**Background:**

Eucalyptus oil poisoning is rare in adults but is not that uncommon in children. The common side effects in children include depression in the level of consciousness, ataxia, seizures, and vomiting. Unlike in children, seizures are unusual in adult patients with eucalyptus oil poisoning. We report the cases of two patients with eucalyptus oil poisoning, both adults who unintentionally took eucalyptus oil and presented to the emergency room of our institution with seizures.

**Case presentation:**

Two adult Indian men who unintentionally consumed eucalyptus oil presented to the emergency room of our institution with seizures. In both patients, arterial blood gas analysis showed the presence of severe metabolic acidosis. Both the patients were managed in the intensive care unit and received standard supportive care. Metabolic acidosis was corrected with intravenous bicarbonate infusion. They were successfully discharged on the fourth day.

**Conclusions:**

All physicians should be aware of the toxic effects of eucalyptus oil, which is used often in daily life in India. Supportive care in an intensive care unit, including rapid correction of metabolic acidosis and adequate maintenance of hemodynamic parameters, will lead to a rapid recovery. Warning labels should be made mandatory on all products that contain eucalyptus oil.

## Background

Eucalyptus oil poisoning is not that uncommon in children [1, 2], and it is usually unintentional [1]. The common side effects in children include depression in the level of consciousness, ataxia, seizures, and vomiting [1, 3]. Intentional or unintentional ingestion of eucalyptus oil is rare in adults. Unlike in children, seizures in adult patients with eucalyptus oil poisoning are unusual [4]. We report the cases of two patients, both adults and intimate friends and living in the same room, who unintentionally consumed eucalyptus oil and presented to the emergency room (ER) of our institution with seizures.

## Case presentation

A 22-year-old Indian man presented to the ER of our institution in a drowsy state at about 9.45 a.m. The patient had been asymptomatic until that morning, when he had one episode of seizure. He was accompanied to the ER by another Indian man, a 26-year-old who was a close friend. Upon further probing of the patient’s history, the friend of the patient revealed that on the same morning, the patient had taken eucalyptus oil, around 15 ml supposedly for abdominal pain. He had bought it in bottle form, and the bottle had the markings of eucalyptus oil written on it but no information regarding precautions or side effects. When it was revealed to the accompanying friend that eucalyptus oil may have been the cause of the patient’s seizure, he also admitted to have consumed the same substance that morning for similar complaints. Soon the accompanying friend also had a bout of seizure while in the ER.

Both the friends were intubated in the ER because of low Glasgow Coma Scale scores and were shifted to the intensive care unit (ICU) for further management. The test results were similar in both patients. Hemograms revealed mild neutrophilic leukocytosis in both patients. The results of renal function and electrolyte panels were normal in both the patients. The patient’s liver enzymes were mildly elevated. The patients' electrocardiograms showed mild tachycardia, and chest radiography was also normal in both patients. Arterial blood gas (ABG) analysis showed the presence of severe metabolic acidosis: patient 1 had a pH of 6.983 (normal range, 7.35–7.45) and HCO_3_^−^ of 8.3 mmol/L (normal range, 24–28 mmol/L); patient 2 had a pH of 7.042 and HCO_3_^−^ of 8.7 mmol/L.

Urine samples of both patients were subjected to toxicological analysis, which confirmed the presence of eucalyptus oil. Gas chromatographic analysis of the sample of eucalyptus oil was also performed, which revealed the presence of α-pinene, myrcene, cineole, fenchone, α-terpinolene, camphor, and β-terpinyl acetate (Table 1).

**Table 1:** Gas chromatographic analysis of the alleged sample eucalyptus oil

No.	Compound	Content (%)
1	α-Pinene	8.28
2	Myrcene	5.86
3	Cineole	27.56
4	Fenchone	3.56
5	α-Terpinolene	2.63
6	Camphor	4.49
7	β-Terpinyl acetate	0.78

Subsequent to orotracheal intubation, both patients underwent gastric lavage. They were managed in the ICU and received standard supportive care. Metabolic acidosis was treated with intravenous bicarbonate infusion, the rate of which was titrated according to the ABG. Both patients also received antiseizure medications.

Metabolic acidosis was corrected in 24–36 hours after admission (Table 2). Bicarbonate infusion was stopped on the second day. Both the patients were successfully weaned off the ventilator and extubated on the second day of admission. They were discharged 2 days later on a tapering regimen of antiseizure medications. They were subsequently lost to follow-up.

**Table 2:** pH and bicarbonate levels (arterial blood gas analysis) of two patients in serial order

Patient 1	Day 1 10:37 a.m.	Day 1 6:00 p.m.	Day 2 5:00 a.m.	Day 2 1:00 p.m.	Day 3 7:00 a.m.	Day 4 6:53 a.m.
pH	6.983	7.3	7.47	7.45	7.38	7.39
HCO_3_^−^	8.3	17.2	18.9	18.1	19.5	24.8
Patient 2	Day 1 12:44 p.m.	Day 1 3:30 p.m.	Day 2 5:00 a.m.	Day 2 4:00 p.m.	Day 3 6:52 a.m.	Day 4 6:50 a.m.
pH	7.04	7.37	7.44	7.37	7.39	7.41
HCO_3_^−^	8.7	15.6	19.7	20.8	22.4	24.1

## Discussion

Eucalyptus oil poisoning is more commonly described in children and is usually unintentional [1, 2]. Intentional or unintentional ingestion of eucalyptus oil in adults is rare. Unlike in children, seizures in adults who have consumed eucalyptus oil are unusual [4]. Our two patients were adults.

α-Pinene, myrcene, cineole, fenchone, α-terpinolene, β-terpinyl acetate, and camphor were the ingredients isolated from the alleged material consumed by our two patients. Of these, α-pinene, myrcene, cineole, fenchone, α-terpinolene, and β-terpinyl acetate are well described in true eucalyptus oil, with cineole being the main constituent [5]. Cineole-based “oil of eucalyptus” is generally considered safe for adults if taken at a low dose, as in the case of flavoring agents or in pharmaceutical products. However, at higher-than-recommended doses, systemic toxicity can occur [6]. Eucalyptus oil taken from the eucalyptus tree (true eucalyptus oil) does not contain camphor. However, the cineole fraction of camphor laurel that is also used to manufacture eucalyptus oil (which is considered “fake eucalyptus oil”) [7] may contain camphor. Like eucalyptus oil, camphor is also epileptogenic [8]. Camphor was also detected in the “eucalyptus oil” that our patients had consumed.

Eucalyptus oil is used as an over-the-counter medication in most countries, in addition to its myriad use in pharmaceutical, flavoring, pesticide, perfumery, and industrial uses. Its permissible limit is often unregulated, however. The use of camphor is similar. Most people, even physicians, are not aware of the toxic potential of these seemingly innocuous substances. Regulation regarding the permissible limits of these ingredients in substances that contain them should be strictly imposed. Also, the label of products that contain eucalyptus oil or camphor should have mandatory warnings of the potential toxic effects, including seizures.

## Conclusions

All physicians should be aware of the toxic effects of eucalyptus oil, which is used often in daily life in India. Supportive care in an ICU, including rapid correction of metabolic acidosis and adequate maintenance of hemodynamic parameters, will lead to a rapid recovery. Warning labels should be made mandatory on all products that contain eucalyptus oil. Our patients cases reflect a poorly documented but severe side effect of a frequently used substance. What makes this case report unusual is the severity and rarity of this complication.

## Data Availability

None.

## Abbreviations

ABG: Arterial blood gas
ER: Emergency room
ICU: Intensive care unit
